# Hepatic abscess induced by gastric cancer mimicking differentiated hepatocellular carcinoma

**DOI:** 10.1002/jgh3.12970

**Published:** 2023-09-19

**Authors:** Takuya Kishimoto, Toshihiro Morita, Shujiro Yazumi

**Affiliations:** ^1^ Department of Gastroenterology and Hepatology Medical Research Institute Kitano Hospital, PIIF Tazuke‐Kofukai Osaka Japan

**Keywords:** gastric cancer, hepatocellular carcinoma, liver abscess

## Abstract

A 58‐year‐old man was referred to our hospital with right abdominal pain. Contrast‐enhanced computed tomography (CECT) showed a rim‐enhanced lesion with a fluid collection in the right hepatic lobe. Upper gastrointestinal endoscopy revealed a Borrmann type 1 tumor on the middle gastric body, identified as an adenocarcinoma on pathology. The patient underwent percutaneous transhepatic abscess drainage and was treated with antibiotics. Two weeks after drainage, CECT revealed shrinkage of the abscess; however, the wall showed contrast enhancement. Needle biopsy was performed for the liver tumor, and it suggested the possibility of highly differentiated hepatocellular carcinoma. The patient first underwent gastrectomy, and the liver tumor was followed with CECT. Two months after surgery, CECT revealed that the liver tumor had vanished. Liver abscesses and infectious tumors can be difficult to differentiate between; therefore, careful scrutiny is essential before treatment.

## Graphical abstract

We experienced a case of *Streptococcus intermedius* related liver abscess induced by gastric cancer. Contrast effect was observed on the tumor wall, hence it was difficult to distinguish it from differentiated hepatocellular carcinoma even after tumor biopsy.

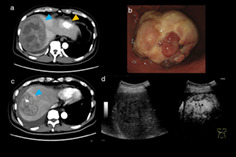

## Introduction

Liver abscess, a fatal inflammatory condition often caused by infectious agents, can be difficult to differentiate from malignant tumors, such as hepatocellular carcinoma or intrahepatic cholangiocarcinoma accompanied with pyogenic infection. *Streptococcus intermedius* stands as an infrequent source of liver abscesses in East Asia while it is frequently associated with gastrointestinal neoplasms, including gastric cancer. Here we report a case of a gastric cancer‐associated liver abscess that was difficult to distinguish from hepatocellular carcinoma, including images and pathological findings.

## Case report

**Figure 1:**
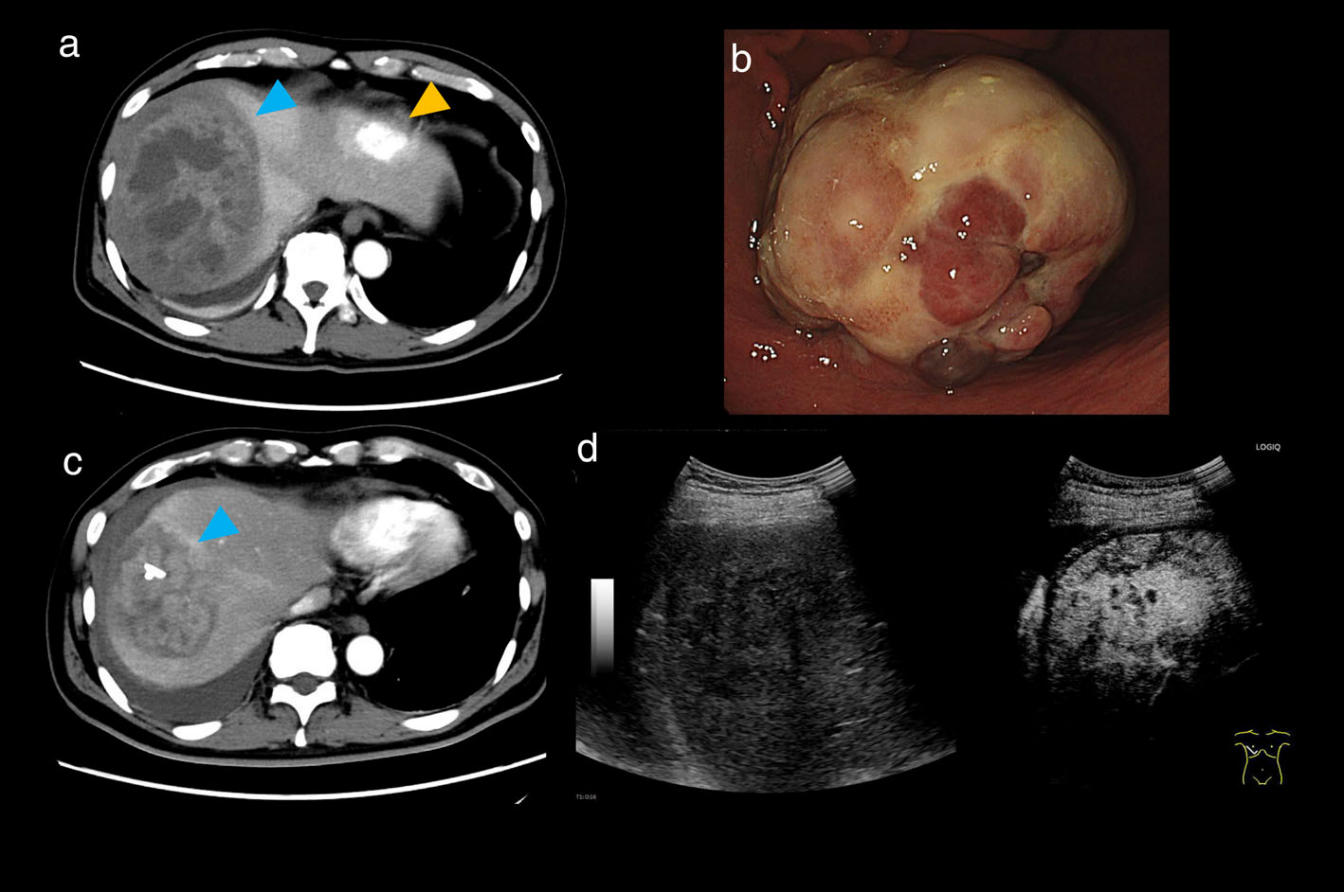
Abdominal contrast‐enhanced computed tomography (CECT) shows a 10‐cm mass with encapsulated fluid in the right hepatic lobe below the diaphragm (a, blue arrowhead) and a hemangioma in the left hepatic lobe (a, yellow arrowhead). Upper gastrointestinal endoscopy imaging shows a contrast‐enhanced 6‐cm‐large mass within the gastric body (b). CECT 2 weeks after drainage shows strong enhancement of the thick septum (c, blue arrowhead). Contrast‐enhanced ultrasonography revealed strong intratumoral enhancement in the arterial phase (d).

A 56‐year‐old man was referred to our hospital with right‐sided abdominal pain. The patient had no relevant medical history. A general examination showed right upper abdominal pain and a high fever (39°C). His blood tests revealed an elevated white blood cell count of 13.9 × 10^3^/μL and an elevated C‐reactive protein level of 16.5 mg/dL. Contrast‐enhanced computed tomography (CECT) revealed a large heterogeneous mass with encapsulated fluid measuring approximately 10 cm in diameter, suggesting a large infectious lesion in the right hepatic lobe below the diaphragm (Fig. 1a, blue arrowhead), a hemangioma in the left hepatic lobe (Fig. 1a, yellow arrowhead), and a contrast‐enhanced 6‐cm mass within the gastric body. Upper gastrointestinal endoscopy revealed a large Borrmann type 1 tumor with depletion of mucosa from almost the entire surface of the tumor on the greater curvature of the middle gastric body (Fig. 1b).

We initially considered the differential diagnoses of a pyogenic liver abscess, metastatic liver tumor, hepatocellular carcinoma (HCC), or intrahepatic cholangiocarcinoma complicated with infection. However, since the patient presented with fever and abdominal pain, we decided that percutaneous drainage was necessary. The patient underwent percutaneous transhepatic abscess drainage with an 8 Fr tube and was prescribed antibiotics. Cytology of the drainage was negative.

Abscess bacteriology revealed *Streptococcus intermedius*, which is indigenous to the oral cavity and gastrointestinal tract. As some reports have shown that *S. intermedius* has induced liver abscesses through the breakdown of the mucosal barrier from gastric cancer,^1^ we thought that this abscess might be induced by gastric cancer.

CECT performed 2 weeks later showed shrinkage of the abscess; however, a strong enhancement of the thick septum was observed (Fig. 1c, blue arrowhead). Contrast‐enhanced ultrasonography revealed strong intratumoral enhancement in the arterial phase (Fig. 1d), suggesting some tumoral region like HCC. 18F‐fluorodeoxyglucose positron emission CT showed no uptake on the tumor wall, which did not positively support a neoplastic lesion such as a metastatic tumor, but the possibility of highly differentiated HCC could not be ruled out.

A needle biopsy of the tumor wall was performed, and the histopathologic examination showed fibrosis with infiltration by lymphocytes and plasma cells, together with some CK7 positive biliary epithelial cells and CD34 positive sinusoidal endothelial cells into the fibrotic tissues, which were not typical of a simple abscess. The pathologist suggested the possibility of a highly differentiated HCC with granulation.

After surgical consultation, the patient initially underwent gastrectomy, and the liver tumor was followed up using CT in order to avoid an excessively invasive hepatectomy.

The final diagnosis of the stomach was poorly differentiated adenocarcinoma (UICC seventh pT3N0M0 stage IIIA). The patient was discharged without any complications and subsequently underwent adjuvant chemotherapy. At his one‐year follow‐up visit, he had no recurrence.

CECT 2 months after gastric surgery showed the disappearance of the liver tumor. Eventually, the granulomatous changes in the abscess wall healed as the abscess healed.

## Discussion

We encountered a case of liver abscess complicated by gastric cancer, which was difficult to distinguish from neoplastic lesions. Pyogenic liver abscess is a fatal infection caused by *Klebsiella pneumoniae* or *Escherichia coli*, mainly in East Asia.^2^ *Streptococcus intermedius* belongs to *Streptococcus anginosus* group (SAG), which is an oral cavity, nasal cavity, pharynx, and gastrointestinal mucosal pathogen often causing brain, lung, and peritoneal abscesses, whereas liver abscess is rare in East Asia. Some cases of liver abscess formation due to the destruction of the mucosal barrier caused by gastrointestinal cancer, similar to the present case, have been reported^3^ recently.

In this case, the loss of mucosa from almost all surfaces of the gastric carcinoma with abundant blood flow, shown as strong contrast enhancement on CECT, may have induced the hematogenous bacterial infection and transportal hepatic abscess.

Given that a malignant neoplasm concomitant with an abscess demonstrates a higher propensity for affiliation with SAG at a rate of 14.3%, as opposed to Klebsiella at a rate of 3.6%,^4^ it is important to conduct an investigation into gastrointestinal lesions in order to rule out malignant tumor in the case of SAG liver abscess.

In addition, metastatic tumors and hepatocellular carcinomas can also complicate liver abscess; therefore, it is necessary to differentiate between abscesses and neoplasms when a liver mass is observed with an associated gastrointestinal carcinoma.

Necrosis of hepatocellular carcinoma with infection and liver abscess are sometimes difficult to differentiate. Yeh *et al*. reported that among 10 cases of HCC accompanied with liver abscess, only five cases were correctly diagnosed before surgical treatment.^5^ Tumor biopsy was effective for the proper diagnosis but sometimes problematic due to false negatives and dissemination.

Since the surgical technique and treatment plan differ greatly depending on the diagnosis of the liver mass, careful preoperative scrutiny is necessary.

In such an instance, it is imperative to entertain the prospect of malignancy and deliberate over the implementation of recurrent imaging investigations and successive biopsy procedures, even subsequent to gastrectomy. While the differentiation between hepatic abscesses and neoplastic formations presents intricate challenges, akin to the scenario at hand, meticulous preoperative assessments inclusive of biopsy procedures assume paramount significance in the selection of an apt therapeutic approach.

### Patient consent statement

Informed consent was obtained from the patient for publication of this case report and accompanying images.
